# Dynamic change of variant allele frequency reveals disease status, clonal evolution and survival in pediatric relapsed B‐cell acute lymphoblastic leukaemia

**DOI:** 10.1002/ctm2.892

**Published:** 2022-05-23

**Authors:** Shuiyan Wu, Lixia Liu, Xinran Chu, Jiajia Zheng, Zixing Chen, Li Gao, Peifang Xiao, Jun Lu, Qi Ji, Jing Ling, Shanbo Cao, Jian Pan, Jiayue Qin, Shaoyan Hu

**Affiliations:** ^1^ Department of Hematology and Oncology Children's Hospital of Soochow University Suzhou China; ^2^ Pediatric Intensive Care Unit Children's Hospital of Soochow University Suzhou China; ^3^ Department of Medical Affairs Acornmed Biotechnology Co., Ltd. Tianjin China; ^4^ Department of Hematology The First Affiliated Hospital of Soochow University Suzhou China; ^5^ Institute of Pediatric Research Children's Hospital of Soochow University Suzhou China

Dear Editor,

Changes in variant allele frequency (VAF) in different mutated genes played an important role in predicting patient disease status and assessing patient prognosis in adult myelodysplastic syndrome and acute myeloid leukaemia,^1^, ^2^, ^3^ but were not well established in pediatric relapsed B‐cell acute lymphoblastic leukaemia (B‐ALL) patients.^4^, ^5^ Thus, we evaluated the mutation profile and dynamic change of VAF in 68 serial bone marrow (BM) samples based on deep targeted next‐generation sequencing (NGS) in 24 Chinese pediatric relapsed B‐ALL patients at diagnosis, remission and relapse, providing insight into predicting disease status, clonal evolution and survival.

The details of clinical data and methods are described in Tables S1 and S2. A total of 71 mutated genes and 204 genetic mutations were detected at diagnosis and/or relapse in the 24 patients (Figure S1 and Table S3). Note that, 90.5% (19/21) of the patients at relapse were often accompanied by new mutations, which is consistent with the results of Malinowska‐Ozdowy et al.^6^ For 21 patients with diagnosis‐relapse pairs, 81.0% (17/21) of the patients had mutations at diagnosis and mutations were detected in all patients at relapse (Figure 1A). Compared with the mutations at diagnosis, the mutations detected at relapse were significantly involved in transcription factor pathway (61.9% vs. 23.8%, *p* = 0.013) (Figure 1B), which plays an essential role in the pathogenesis of pediatric ALL.^7^, ^8^ Moreover, the number of mutations in pediatric B‐ALL patients at relapse tended to be higher than that at diagnosis (median 3 vs. 2, *p *= 0.080), indicating that the mutation profile of B‐ALL patients at relapse is more complex. We further examined the mutation types and no significant difference was shown in the distribution of conversion and transversion mutations between the two time points (*p* = 0.843) (Figure S2).

**FIGURE 1:**
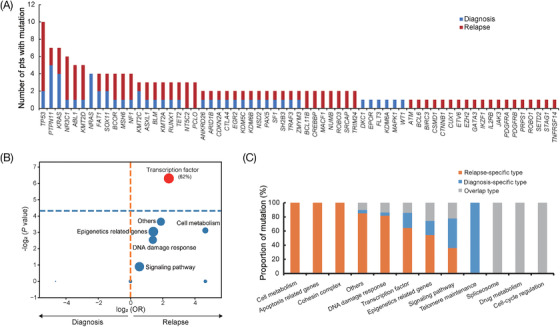
Comparative analysis of mutation distribution in 21 patients with diagnosis‐relapse paired samples. (A) Histogram showing the comparison of mutated genes at diagnosis and relapse. Mutation at diagnosis and relapse is colored blue and red, respectively. (B) Volcano plot showing the comparison of functional pathways in which the mutated genes are involved at diagnosis and relapse. The horizontal axis represents the magnitude of association (log_2_ odds ratio), and the vertical axis indicates the ‐log_2_ *p‐*value. Each circle shows a functional pathway and the size of each circle indicates the frequency of the mutated gene involved in functional pathways. (C) Distribution of three mutation types, including diagnosis‐specific type (only present at diagnosis), relapse‐specific type (only present at relapse) and overlap type (present at both diagnosis and relapse) according to different pathways

Based on the 153 mutation sites at diagnosis and/or relapse, we categorized mutation sites into three types, including diagnosis‐specific type (only present at diagnosis), relapse‐specific type (only present at relapse) and overlap type (present both at diagnosis and relapse). According to the enrichment analysis of mutation sites involved in different gene pathways, diagnosis‐specific type, relapse‐specific type and overlap type were the most common in telomere maintenance pathway, cell metabolism/apoptosis‐related genes/cohesion complex pathway and spliceosome/drug metabolism/cell cycle regulation pathway, respectively, suggesting that these functional pathways play an important role in relapsed B‐ALL (Figure 1C).

We next investigated VAF changes with serial NGS assessments and discovered delta VAF from diagnosis was significantly associated with response to treatment. In 15 patients with diagnosis‐remission pairs, patients achieving complete remission (CR) had significant VAF reduction (*p *< 0.001) (Figure 2A), revealing that several potential markers could be used for detecting minimal residual disease (MRD), including *TP53*, *NRAS*, *PTPN11* and *NF1*, as these mutations disappeared in CR. Moreover, in 18 patients with remission‐relapse pairs, VAF was significantly increased in relapsed patients, indicating delta VAF can be used to evaluate patients for recurrence (*p *< 0.001) (Figure 2B).

**FIGURE 2:**
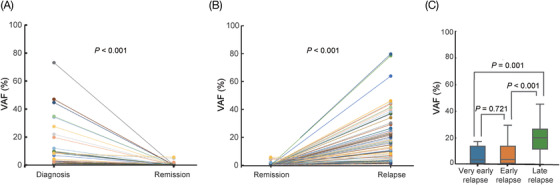
Dynamic change of variant allele frequency (VAF) with serial next‐generation sequencing (NGS) assessments. (A) Comparison of VAFs at diagnosis and remission in 15 patients with diagnosis‐remission paired samples. (B) Comparison of VAFs at remission and relapse in 18 patients with remission‐relapse paired samples. (C) Comparison of VAFs at three different relapse stages in 24 relapsed patients, including very early, early and late relapse stage. Each point in different color represents an independent VAF mutation site. VAF, variant allele frequency; NGS, next‐generation sequencing

Depending on the time of relapse, we divided patients into three groups, including very early (<18 months), early (18–36 months) and late (>36 months) relapse stages.^9^ We found that the VAF in the late relapse stage was significantly higher than that in both very early and early relapse stages (*p *= 0.001; *p *< 0.001), while no statistical difference was discovered in VAF between the very early and early stages (*p *= 0.721, Figure 2C), suggesting that continuous gene mutation monitoring during the CR period would facilitate the early detection of clones, and early clinical intervention might help to prolong the CR time.

Clonal evolution diagrams were produced based on the mutations. We found three relapse‐related clonal evolution patterns, including relapse evolving from a genetically distinct clone (Pattern A), a major clone at diagnosis (Pattern B), and a subclone at diagnosis (Pattern C), respectively (Figure 3). For patient 19 (P19), belonging to Pattern A, flow cytometry showed MRD negative with 3% of BM blasts, and the genetic results showed lower VAF mutations in the *TP53*, *PTPN11* and *PAX5* genes at a four‐month time point prior to relapse (Figure 3B,C). Compared with the genetic results at relapse, we found that the novel enlarged clonal mutation led to the eventual relapse, suggesting serial gene mutation detection in CR period is helpful for early detection of molecular recurrence. However, this phenomenon was not observed in P16 and P18. For P16, belonging to Pattern B, *TP53* p.R248Q mutation was detected at initial diagnosis, disappeared at remission, 2.5 months after remission, 1.5 months before relapse, and 1 month before relapse, and reappeared at relapse (Figure 3B,C), suggesting that *TP53* is an effective therapeutic monitoring indicator. For P18, belonging to Pattern C, had mutations in *KRAS* p.A146V, *PTPN11* p.E76K and *MAPK1* p.7_8del that were cleared after treatment, but the mutation in *PTPN11* p.D61H expanded at relapse (2.9% vs. 21.6%), indicating that the proportion of *RAS* signaling pathway subclones might be reduced during treatment, expanded in the later stage and caused relapse (Figure 3B,C), which is consistent with the results of Ma et al.^10^

**FIGURE 3:**
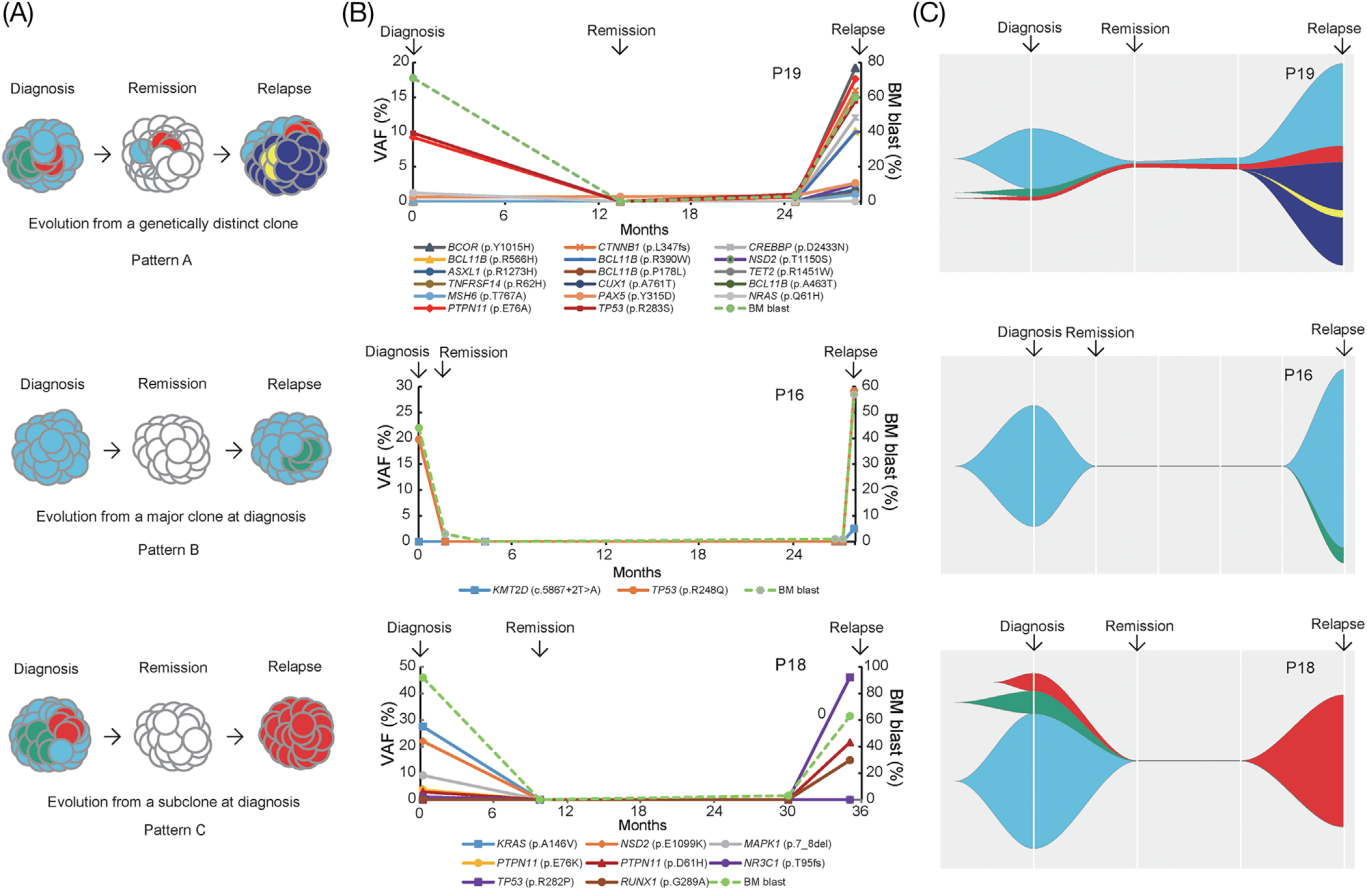
Relapse‐related clonal evolution patterns based on variant allele frequency (VAF). (A) Schematic diagram of three relapse‐related clonal evolution patterns, including relapse evolution from a genetically distinct clone, a major clone at diagnosis, and a subclone at diagnosis, named clonal evolution patterns A, B and C, respectively. Longitudinal analysis of VAF (B) and relapse‐related clonal evolution pattern (C) in three representative pediatric B‐ALL patients. The horizontal axis represents follow‐up time, and the vertical axis indicates VAF. Each point in different color represents an independent VAF mutation site. VAF, variant allele frequency; BM, bone marrow

In order to explore the prognostic value of VAF, we analyzed the association between VAF at relapse and survival. Based on the maximally selected log‐rank statistic, mean VAF was found to be the cutoff point for differentiating patient prognosis. Compared with patients carrying a lower mutation load (mean VAF < 20%), patients with a higher mutation load (mean VAF ≥ 20%) had poorer OS (median survival 3.7 vs. 14.1 months, *p* = 0.032) (Figure 4A) and OS censored at transplantation (median survival 2.0 vs. 18.9 months, *p* = 0.022) (Figure 4B), which suggests that mean VAF at relapse can stratify the prognosis of relapsed patients.

**FIGURE 4:**
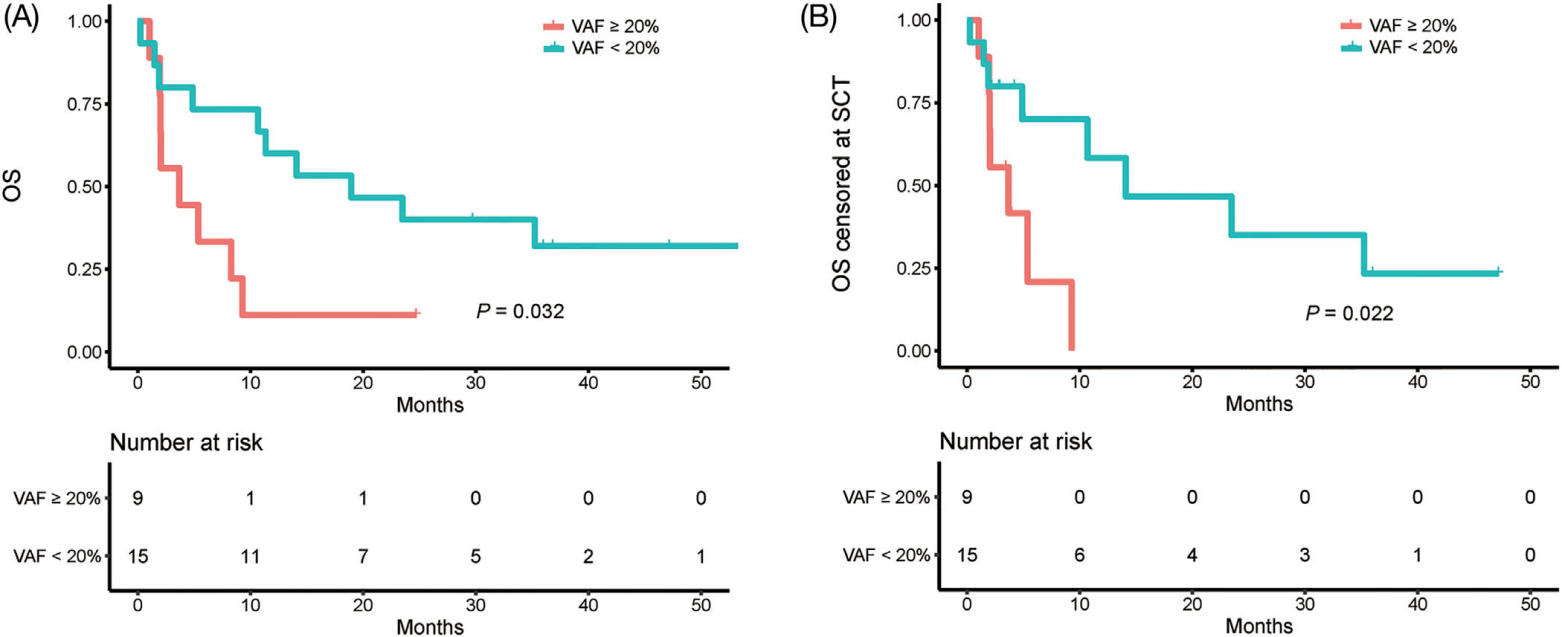
Prognostic impact of variant allele frequency (VAF) on survival. Overall survival (OS) (A) and OS censored at the time of transplantation (B) of 24 relapsed pediatric B‐ALL patients with mean VAF ≥20% versus patients with mean VAF < 20% at relapse. VAF, variant allele frequency; OS, overall survival; SCT, transplantation

In conclusion, our study highlights that dynamic change of VAF reveals the disease status, clonal evolution, and survival in pediatric relapsed B‐ALL patients, and serial molecular detections during the remission period contribute to early detection of relapse, which plays an important role in early intervention and precision therapy.

## CONFLICT OF INTEREST

The authors declare no conflict of interest.

## Supporting information



Supporting Information
