# Supplementary material for: Dynamic change of variant allele frequency reveals disease status, clonal evolution and survival in pediatric relapsed B‐cell acute lymphoblastic leukaemia
Source: Clin Transl Med. 2022 May 23;12(5):e892. doi: 10.1002/ctm2.892 (PMC9126496; doi:10.1002/ctm2.892)
Supplement: Supplementary file 1 — Supporting Information [file CTM2-12-e892-s001.docx]

**Supplementary Information**

**Dynamic change of variant allele frequency reveals disease status, clonal evolution and survival in pediatric relapsed B-cell acute lymphoblastic leukemia**

Shuiyan Wu^1,2*^, Lixia Liu^3*^, Xinran Chu^1*^, Jiajia Zheng^1*^, Zixing Chen^4^, Li Gao^1^, Peifang Xiao^1^, Jun Lu^1^, Qi Ji^1^, Jing Ling^1^, Shanbo Cao^3^, Jian Pan^5^, Jiayue Qin^3^, Shaoyan Hu^1^

^1^ Department of Hematology and Oncology, Children's Hospital of Soochow University, Suzhou, China.

^2^ Pediatric Intensive Care Unit, Children's Hospital of Soochow University, Suzhou, China.

^3^ Department of Medical Affairs, Acornmed Biotechnology Co., Ltd., Tianjin, China.

^4^ Department of Hematology, The First Affiliated Hospital of Soochow University, Suzhou, China.

^5^ Institute of Pediatric Research, Children’s Hospital of Soochow University, Suzhou, China.

^*^These authors contributed equally.

**Correspondence:** Prof. Shaoyan Hu, Department of Hematology and Oncology, Children's Hospital of Soochow University, No. 92, Zhongnan Street, Suzhou 215002, China. E-mail: [hushaoyan@suda.edu.cn](mailto:hushaoyan@suda.edu.cn); Prof. Jiayue Qin, Department of Medical Affairs, Acornmed Biotechnology Co., Ltd., Building D4, International Enterprise Community, Changyuan Road, Wuqing District, Tianjin 301799, China. E-mail: [jyqin@live.cn](mailto:jyqin@live.cn).

This file includes:

Materials and Methods

Figure S1 to S2

Table S1 to S3

**Materials and Methods**

**Patients**

A total of 24 relapsed patients with pediatric B-ALL from the Children’s Hospital of Soochow University were enrolled in this study. All patients and/or their parents/guardians provided signed consent before enrolment. The study was approved by the Children’s Hospital of Soochow University Institutional Review Board in accordance with the Declaration of Helsinki.

Morphological, immunophenotypic and cytogenetic analyses were performed at diagnosis and relapse. Two chemotherapeutic regimens were carrying out: 7 patients were treated with the CCLG 2008 protocol modified from BFM ALL-protocol and the remaining 17 patients were treated with the CCCG 2015 protocol modified from TXV protocol of St. Jude Hospital. According to the criteria of each protocol, patients were classified into low-risk (LR), intermediate-risk (IR) or high-risk (HR). The time of relapse from diagnosis was divided into three phases, including very early (<18 months), early (18-36 months) and late (>36 months) relapse stages.

**Next-generation sequencing**

Genomic DNA was extracted from BM samples collected at the time of diagnosis, remission and relapse from 24 patients, including 15 diagnosis-remission-relapse trios, 6 diagnosis-relapse pairs and 3 remission-relapse pairs. Gene library amplification was performed using KAPA Hyper Prep Kit and 185-gene sequencing panel from Acornmed Biotechnology Co., Ltd was used to capture the target regions through the Illumina Novaseq platform (Table S2).

The following criteria were used to filter raw variant results: average effective sequencing depth on target per sample ≥1,000x, mapping quality ≥30 and base quality ≥30. Burrows-Wheeler Alignment tool (BWA, version 0.7.12) was used to align the trimmed reads. MarkDuplicates tool from Picard was performed to mark PCR duplicates. IndelRealigner and BaseRecalibrator from Genome Analysis Toolkit (GATK, version 3.8) were used for realignment and recalibration of the BWA alignment results, respectively. Mutect2 was used for identifying single nucleotide variations (SNVs) and insertions or deletions (Indels). All the variants were annotated by ANNOVAR software using the resources, including 1000G projects, COSMIC, SIFT and Polyphen. Mutated genes with VAF ≥ 0.5% for SNVs and Indels were included in the analysis. Based on the chromosome ploidy correction and hypothesis that mutation frequencies were under binomial distribution, Acorndx clonal evolution algorithm, made in house, was used to cluster mutations and calculate the possibility whether mutations originate from the same kind of cells, using by hierarchical Bayesian statistical model and Dirichlet process.

**Statistical analysis**

Statistical analysis was performed using SPSS software version 22.0 or R version 3.5.2. Categorical variables were compared using chi-square test, or Fisher’s exact test as appropriate. Continuous variables were compared using the non-parametric Mann-Whitney or Wilcoxon test when appropriate. Survival analyses were evaluated utilizing the Kaplan-Meier method and curves compared via the log-rank test. Overall survival (OS) was calculated from relapse to death or last follow-up. A two-sided *P* value < 0.05 was considered to indicate a statistical significance.


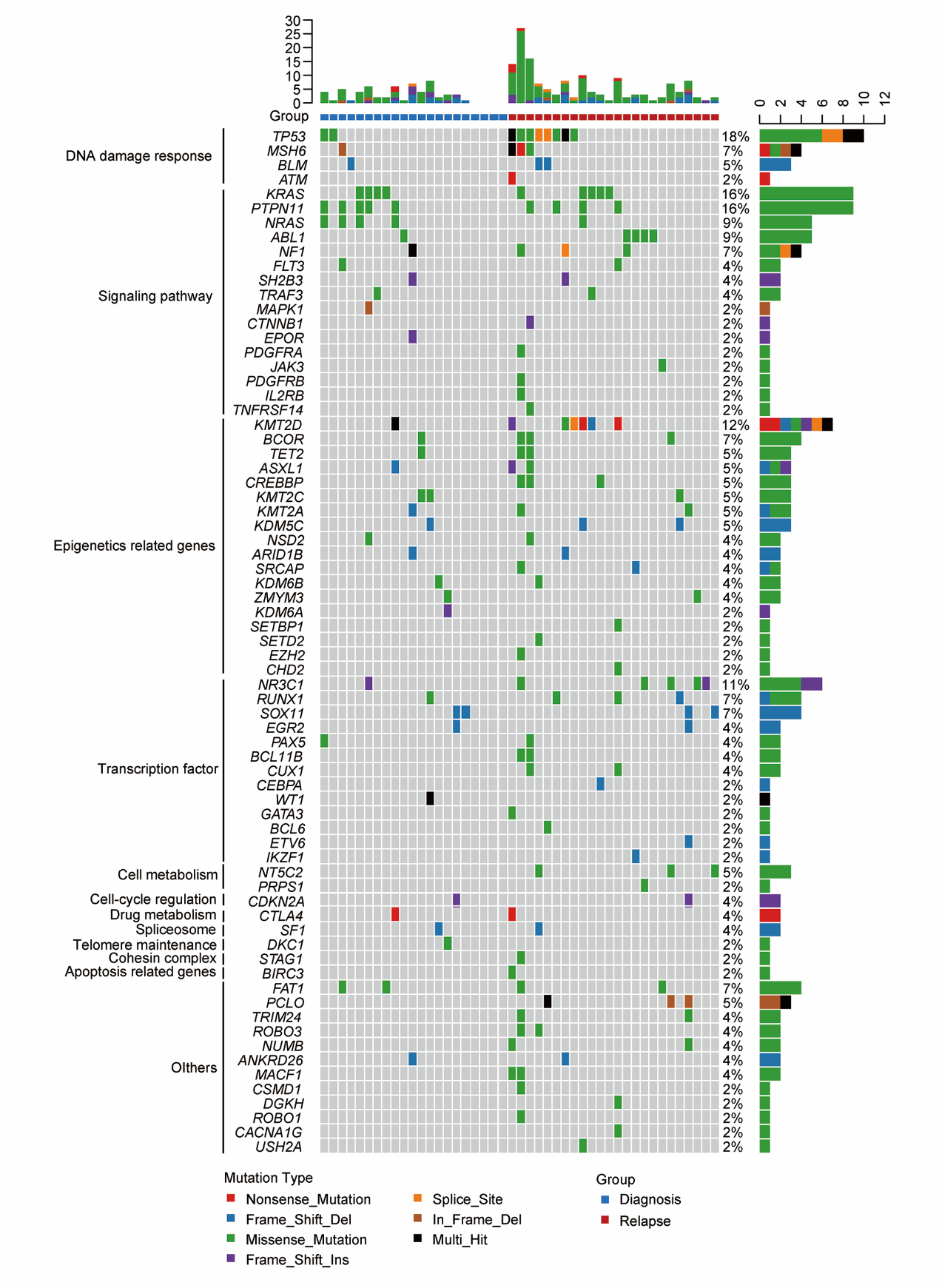


**Figure S1.** Mutation landscape in 24 pediatric B-ALL patients.


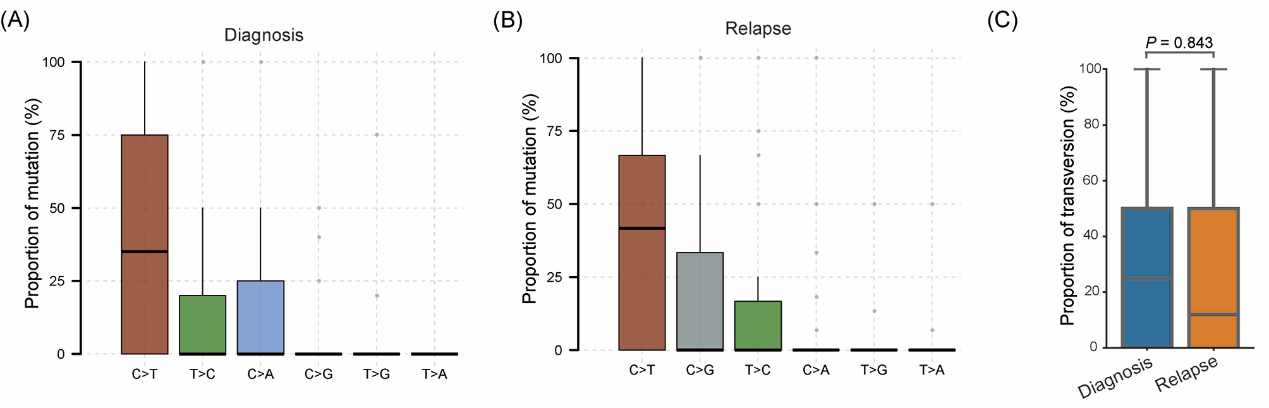


**Figure S2.** Comparison of conversion and transversion mutations at diagnosis and relapse in 21 pediatric B-ALL patients with diagnosis-relapse paired samples.

**Table S1. Clinical and biological features of pediatric relapsed B-ALL patients**

| Patient | Sex | Age at diagnosis | Immunophenotype at diagnosis | Fusion gene at diagnosis | Immunophenotype at relapse | Risk stratification | Relapse site | Relapse stage | Transplantation | Survival |
| --- | --- | --- | --- | --- | --- | --- | --- | --- | --- | --- |
| P1 | M | 5Y1M | B | Negative | B | IR | BM | Late | No | Dead |
| P2 | F | 7Y8M | B | Negative | B | HR | BM | Late | No | Dead |
| P3 | F | 13Y4M | B | Negative | B | IR | BM | Late | No | Dead |
| P4 | F | 2Y9M | B | Negative | B | IR | BM | Early | No | Dead |
| P5 | M | 2Y5M | B and myeloid | Negative | B and T | HR | BM | Early | Yes | Live |
| P6 | F | 8Y3M | B | TEL-AML1 | B | LR | BM | Early | Yes | Live |
| P7 | M | 8Y9M | B | Negative | B | IR | BM+CNS | Late | No | Dead |
| P8 | M | 10Y4M | B | Negative | B | IR | BM | Late | Yes | Live |
| P9 | F | 8Y2M | B | Negative | B | LR | BM | Very early | No | Dead |
| P10 | M | 4Y5M | B | BCR-ABL | B | IR | BM+CNS | Very early | No | Dead |
| P11 | F | 8Y10M | B | BCR-ABL | B | IR | BM | Early | Yes | Live |
| P12 | F | 6Y2M | B | TEL-AML1 | B | LR | BM | Very early | No | Dead |
| P13 | M | 8Y8M | B | E2A-PBX1 | B | IR | BM | Early | No | Live |
| P14 | M | 11Y11M | B | BCR-ABL | B | IR | BM | Very early | No | Dead |
| P15 | F | 1Y11M | B | Negative | B | IR | BM | Late | No | Dead |
| P16 | F | 9Y1M | B | MLL-AF4 | B | IR | BM | Early | No | Live |
| P17 | M | 5Y3M | B | Negative | B | IR | BM | Late | Yes | Live |
| P18 | M | 9Y6M | B | Negative | B | IR | BM | Late | Yes | Live |
| P19 | M | 3Y9M | B | Negative | B | IR | BM | Early | No | Live |
| P20 | M | 6Y9M | B | BCR-ABL | B | IR | BM | Early | Yes | Live |
| P21 | M | 2Y5M | B | Negative | B | IR | BM | Very early | No | Live |
| P22 | F | 4Y7M | B | Negative | B | LR | BM | Very early | No | Dead |
| P23 | F | 6Y1M | B | Negative | B | LR | BM | Very early | Yes | Dead |
| P24 | M | 4Y7M | B | Negative | B | IR | BM | Very early | No | Dead |

F, female; M, male; Y, years; M: months; B, B-cell; T, T-cell; LR, low-risk; IR, intermediate-risk; HR, high-risk; BM, bone marrow; CNS, central nervous system.

**Table S2. The 185-gene sequencing panel**

| **Gene** | **Transcript** | **Pathway** |
| --- | --- | --- |
| ABCB1 | NM_000927 | Drug metabolism |
| ABCG2 | NM_001257386 | Drug metabolism |
| ABL1 | NM_005157 | Signaling pathway |
| ADSL | NM_000026 | Drug metabolism |
| ALK | NM_004304 | Signaling pathway |
| ANKRD26 | NM_001256053 | Others |
| ARID1B | NM_017519 | Epigenetics related genes |
| ASNS | NM_001178075 | Others |
| ASXL1 | NM_015338 | Epigenetics related genes |
| ASXL2 | NM_018263 | Epigenetics related genes |
| ATM | NM_000051 | DNA damage response |
| ATRX | NM_000489 | Epigenetics related genes |
| B2M | NM_004048 | Immune escape |
| BCL11B | NM_138576 | Transcription factor |
| BCL2 | NM_000633 | Apoptosis related genes |
| BCL6 | NM_001706 | Transcription factor |
| BCOR | NM_001123383 | Epigenetics related genes |
| BCORL1 | NM_021946 | Epigenetics related genes |
| BIRC3 | NM_001165 | Apoptosis related genes |
| BLM | NM_000057 | DNA damage response |
| BMP7 | NM_001719 | Signaling pathway |
| BRAF | NM_004333 | Signaling pathway |
| BTK | NM_000061 | Signaling pathway |
| CACNA1G | NM_018896 | Others |
| CALR | NM_004343 | Others |
| CBL | NM_005188 | Signaling pathway |
| CCDC168 | NM_001146197 | Others |
| CCND3 | NM_001760 | Cell-cycle regulation |
| CD79A | NM_001783 | Signaling pathway |
| CDA | NM_001785 | Drug metabolism |
| CDKN1B | NM_004064 | Cell-cycle regulation |
| CDKN2A | NM_000077 | Cell-cycle regulation |
| CEBPA | NM_004364 | Transcription factor |
| CECR2 | NM_031413 | Epigenetics related genes |
| CEP72 | NM_018140 | Drug metabolism |
| CHD2 | NM_001271 | Epigenetics related genes |
| CPA2 | NM_001869 | Drug metabolism |
| CREBBP | NM_004380 | Epigenetics related genes |
| CRLF2 | NM_022148 | Signaling pathway |
| CSF3R | NM_156039 | Signaling pathway |
| CSMD1 | NM_033225 | Others |
| CTCF | NM_006565 | Epigenetics related genes |
| CTLA4 | NM_005214 | Drug metabolism |
| CTNNB1 | NM_001904 | Signaling pathway |
| CUX1 | NM_181552 | Transcription factor |
| CYP3A5 | NM_000777 | Drug metabolism |
| CYPA | NM_001300981 | Others |
| DARS | NM_145507 | Drug metabolism |
| DDX41 | NM_016222 | Others |
| DGKH | NM_001204504 | Others |
| DHX15 | NM_001358 | Spliceosome |
| DHX30 | NM_138615 | Spliceosome |
| DIS3 | NM_014953 | Others |
| DKC1 | NM_001363 | Telomere maintenance |
| DNAH2 | NM_020877 | Others |
| DNM2 | NM_001005361 | Others |
| DNMT3A | NM_022552 | Epigenetics related genes |
| DOK5 | NM_001294161 | Drug metabolism |
| DROSHA | NM_001100412 | Drug metabolism |
| EGR2 | NM_000399 | Transcription factor |
| ELANE | NM_001972 | Others |
| EP300 | NM_001429 | Epigenetics related genes |
| EPOR | NM_000121 | Signaling pathway |
| ERCC1 | NM_001166049 | Drug metabolism |
| ETNK1 | NM_018638 | Cell metabolism |
| ETV6 | NM_001987 | Transcription factor |
| EZH2 | NM_001203247 | Epigenetics related genes |
| FAM46C | NM_017709 | Others |
| FAT1 | NM_005245 | Others |
| FBXW7 | NM_033632 | Signaling pathway |
| FCGR3A | NM_000569 | Drug metabolism |
| FLT3 | NM_004119 | Signaling pathway |
| FOXO1 | NM_002015 | Transcription factor |
| GART | NM_001136006 | Others |
| GATA1 | NM_002049 | Transcription factor |
| GATA2 | NM_032638 | Transcription factor |
| GATA3 | NM_002051 | Transcription factor |
| GFI1 | NM_005263 | Transcription factor |
| GNAS | NM_080425 | Signaling pathway |
| GRIA1 | NM_000827 | Others |
| GSTM1 | NM_000561 | Drug metabolism |
| GSTP1 | NM_000852 | Drug metabolism |
| ID3 | NM_002167 | Others |
| IDH1 | NM_001282386 | Epigenetics related genes |
| IDH2 | NM_002168 | Epigenetics related genes |
| IKZF1 | NM_006060 | Transcription factor |
| IL2RB | NM_001346223 | Signaling pathway |
| IL7R | NM_002185 | Signaling pathway |
| IMPDH2 | NM_000884 | Drug metabolism |
| ITPA | NM_001267623 | Drug metabolism |
| JAK1 | NM_002227 | Signaling pathway |
| JAK2 | NM_004972 | Signaling pathway |
| JAK3 | NM_000215 | Signaling pathway |
| KDM5C | NM_004187 | Epigenetics related genes |
| KDM6A | NM_001291415 | Epigenetics related genes |
| KDM6B | NM_001080424 | Epigenetics related genes |
| KIT | NM_000222 | Signaling pathway |
| KMT2A | NM_001197104 | Epigenetics related genes |
| KMT2C | NM_170606 | Epigenetics related genes |
| KMT2D | NM_003482 | Epigenetics related genes |
| KRAS | NM_004985 | Signaling pathway |
| MACF1 | NM_012090 | Others |
| MAP2K1 | NM_002755 | Signaling pathway |
| MAPK1 | NM_002745 | Signaling pathway |
| MED12 | NM_005120 | Signaling pathway |
| MPL | NM_005373 | Signaling pathway |
| MSH6 | NM_000179 | DNA damage response |
| MTHFR | NM_001330358 | Drug metabolism |
| MTRR | NM_001364440 | Drug metabolism |
| MYC | NM_002467 | Transcription factor |
| MYD88 | NM_002468 | Signaling pathway |
| NF1 | NM_000267 | Signaling pathway |
| NFATC2 | NM_001136021 | Drug metabolism |
| NOTCH1 | NM_017617 | Signaling pathway |
| NPM1 | NM_002520 | Others |
| NR3C1 | NM_000176 | Transcription factor |
| NRAS | NM_002524 | Signaling pathway |
| NSD2 | NM_133330 | Epigenetics related genes |
| NT5C2 | NM_012229 | Cell metabolism |
| NUDT15 | NM_001304745 | Drug metabolism |
| NUMB | NM_001005743 | Others |
| PAX5 | NM_016734 | Transcription factor |
| PCLO | NM_033026 | Others |
| PDGFRA | NM_006206 | Signaling pathway |
| PDGFRB | NM_002609 | Signaling pathway |
| PHF6 | NM_001015877 | Epigenetics related genes |
| PIGA | NM_002641 | Cell metabolism |
| PIK3R1 | NM_181523 | Signaling pathway |
| PLCG2 | NM_002661 | Signaling pathway |
| PNPLA3 | NM_025225 | Drug metabolism |
| PPM1D | NM_003620 | DNA damage response |
| PRKDC | NM_006904 | DNA damage response |
| PRPF8 | NM_006445 | Spliceosome |
| PRPS1 | NM_002764 | Cell metabolism |
| PTEN | NM_000314 | Signaling pathway |
| PTPN11 | NM_002834 | Signaling pathway |
| RAD21 | NM_006265 | Cohesin complex |
| RB1 | NM_000321 | Cell-cycle regulation |
| ROBO1 | NM_002941 | Others |
| ROBO3 | NM_022370 | Others |
| RPL10 | NM_006013 | Ribosome related genes |
| RRM1 | NM_001033 | Drug metabolism |
| RRM2 | NM_001034 | Drug metabolism |
| RRM2B | NM_001172477 | Drug metabolism |
| RUNX1 | NM_001754 | Transcription factor |
| SERPINE1 | NM_000602 | Drug metabolism |
| SETBP1 | NM_015559 | Epigenetics related genes |
| SETD2 | NM_014159 | Epigenetics related genes |
| SF1 | NM_201995 | Spliceosome |
| SF3B1 | NM_012433 | Spliceosome |
| SH2B3 | NM_005475 | Signaling pathway |
| SLC22A1 | NM_003057 | Drug metabolism |
| SLCO1A2 | NM_001386878 | Drug metabolism |
| SLCO1B1 | NM_006446 | Drug metabolism |
| SMC1A | NM_006306 | Cohesin complex |
| SMC3 | NM_005445 | Cohesin complex |
| SOD2 | NM_000636 | Drug metabolism |
| SOS1 | NM_005633 | Signaling pathway |
| SOX11 | NM_003108 | Transcription factor |
| SPI1 | NM_003120 | Transcription factor |
| SRCAP | NM_006662 | Epigenetics related genes |
| SRGAP1 | NM_001346201 | Others |
| SRGAP2 | NM_001170637 | Others |
| SRGAP3 | NM_001033117 | Others |
| SRP72 | NM_006947 | Others |
| SRSF2 | NM_003016 | Spliceosome |
| STAG1 | NM_005862 | Cohesin complex |
| STAG2 | NM_001042749 | Cohesin complex |
| STAT3 | NM_003150 | Signaling pathway |
| STAT5B | NM_012448 | Signaling pathway |
| TET2 | NM_001127208 | Epigenetics related genes |
| TNF | NM_000594 | Drug metabolism |
| TNFAIP3 | NM_006290 | Signaling pathway |
| TNFRSF14 | NM_003820 | Signaling pathway |
| TP53 | NM_000546 | DNA damage response |
| TRAF3 | NM_003300 | Signaling pathway |
| TRIM24 | NM_003852 | Others |
| U2AF1 | NM_006758 | Spliceosome |
| USH2A | NM_206933 | Others |
| USP7 | NM_003470 | Others |
| VEGFC | NM_005429 | Others |
| WT1 | NM_024426 | Transcription factor |
| XRCC5 | NM_021141 | Drug metabolism |
| ZMYM3 | NM_001171162 | Epigenetics related genes |
| ZRSR2 | NM_005089 | Spliceosome |

| **Table S3. Mutation sites detected in 24 pediatric B-ALL patients** | | | | | | | |
| --- | --- | --- | --- | --- | --- | --- | --- |
| **Patient** | **Time** | **Gene** | **cHGVS** | **pHGVS** | **MutFreq** | **Transcript** | **ExonicFunc_refGene** |
| P1 | relapse | KMT2D | c.14710C>T | p.R4904X | 0.0111 | NM_003482 | stopgain |
| P1 | relapse | FLT3 | c.1775T>C | p.V592A | 0.4159 | NM_004119 | nonsynonymous SNV |
| P1 | relapse | CHD2 | c.1862G>A | p.R621Q | 0.0107 | NM_001271 | nonsynonymous SNV |
| P1 | relapse | SETBP1 | c.1951A>G | p.K651E | 0.2013 | NM_015559 | nonsynonymous SNV |
| P1 | relapse | PTPN11 | c.214G>A | p.A72T | 0.2366 | NM_002834 | nonsynonymous SNV |
| P1 | relapse | DGKH | c.2827G>A | p.A943T | 0.2497 | NM_001204504 | nonsynonymous SNV |
| P1 | relapse | CUX1 | c.4396G>A | p.A1466T | 0.2647 | NM_181552 | nonsynonymous SNV |
| P1 | relapse | RUNX1 | c.493G>A | p.G165S | 0.1299 | NM_001754 | nonsynonymous SNV |
| P1 | relapse | CACNA1G | c.5404G>A | p.D1802N | 0.2227 | NM_018896 | nonsynonymous SNV |
| P2 | diagnosis | KMT2D | c.10360C>T | p.Q3454X | 0.3483 | NM_003482 | stopgain |
| P2 | diagnosis | ASXL1 | c.1927dupG | p.G642fs | 0.027 | NM_015338 | frameshift insertion |
| P2 | diagnosis | PTPN11 | c.214G>A | p.A72T | 0.0866 | NM_002834 | nonsynonymous SNV |
| P2 | diagnosis | NRAS | c.35G>T | p.G12V | 0.3433 | NM_002524 | nonsynonymous SNV |
| P2 | diagnosis | KMT2D | c.3919_3920insACCC | p.S1307fs | 0.0931 | NM_003482 | frameshift insertion |
| P2 | diagnosis | CTLA4 | c.66C>A | p.C22X | 0.1195 | NM_005214 | stopgain |
| P2 | relapse | ATM | c.1369C>T | p.R457X | 0.2495 | NM_000051 | stopgain |
| P2 | relapse | BIRC3 | c.155G>A | p.R52H | 0.108 | NM_001165 | nonsynonymous SNV |
| P2 | relapse | ASXL1 | c.1927dupG | p.G642fs | 0.1598 | NM_015338 | frameshift insertion |
| P2 | relapse | MSH6 | c.3656C>A | p.T1219N | 0.302 | NM_000179 | nonsynonymous SNV |
| P2 | relapse | MSH6 | c.366_367insAA | p.E122fs | 0.2381 | NM_000179 | frameshift insertion |
| P2 | relapse | MACF1 | c.4049A>G | p.Y1350C | 0.2625 | NM_012090 | nonsynonymous SNV |
| P2 | relapse | TP53 | c.473G>A | p.R158H | 0.0711 | NM_000546 | nonsynonymous SNV |
| P2 | relapse | TP53 | c.586C>T | p.R196X | 0.0318 | NM_000546 | stopgain |
| P2 | relapse | NUMB | c.62G>A | p.R21H | 0.2016 | NM_001005743 | nonsynonymous SNV |
| P2 | relapse | CTLA4 | c.66C>A | p.C22X | 0.4353 | NM_005214 | stopgain |
| P2 | relapse | TP53 | c.743G>A | p.R248Q | 0.1453 | NM_000546 | nonsynonymous SNV |
| P2 | relapse | KMT2D | c.7996_7997insC | p.D2666fs | 0.3853 | NM_003482 | frameshift insertion |
| P2 | relapse | TP53 | c.817C>T | p.R273C | 0.1799 | NM_000546 | nonsynonymous SNV |
| P2 | relapse | GATA3 | c.893G>A | p.R298Q | 0.2271 | NM_002051 | nonsynonymous SNV |
| P3 | diagnosis | KDM6A | c.2658dupG | p.E886fs | 0.0167 | NM_001291415 | frameshift insertion |
| P3 | diagnosis | ZMYM3 | c.2741A>G | p.N914S | 0.4697 | NM_001171162 | nonsynonymous SNV |
| P3 | diagnosis | DKC1 | c.863C>G | p.P288R | 0.0221 | NM_001363 | nonsynonymous SNV |
| P3 | relapse | NR3C1 | c.1691A>G | p.N564S | 0.7852 | NM_000176 | nonsynonymous SNV |
| P3 | relapse | ZMYM3 | c.2741A>G | p.N914S | 0.3408 | NM_001171162 | nonsynonymous SNV |
| P4 | remission | CEBPA | c.564_566del | p.188_189del | 0.043 | NM_004364 | frameshift deletion |
| P4 | relapse | KRAS | c.35G>A | p.G12D | 0.1451 | NM_004985 | nonsynonymous SNV |
| P4 | relapse | CREBBP | c.4305T>A | p.D1435E | 0.415 | NM_004380 | nonsynonymous SNV |
| P4 | relapse | CEBPA | c.564_566del | p.188_189del | 0.0551 | NM_004364 | frameshift deletion |
| P5 | diagnosis | WT1 | c.1102delG | p.V368fs | 0.1123 | NM_024426 | frameshift deletion |
| P5 | diagnosis | WT1 | c.1138C>G | p.R380fs | 0.0104 | NM_024426 | frameshift insertion |
| P5 | diagnosis | WT1 | c.1142_1143insCCCC | p.S381fs | 0.0735 | NM_024426 | frameshift insertion |
| P5 | diagnosis | WT1 | c.1385G>A | p.R462Q | 0.0162 | NM_024426 | nonsynonymous SNV |
| P5 | diagnosis | KMT2C | c.2185A>G | p.N729D | 0.086 | NM_170606 | nonsynonymous SNV |
| P5 | diagnosis | KMT2C | c.2189C>A | p.S730Y | 0.03 | NM_170606 | nonsynonymous SNV |
| P5 | diagnosis | RUNX1 | c.320G>A | p.R107H | 0.6683 | NM_001754 | nonsynonymous SNV |
| P5 | diagnosis | KDM5C | c.4378_4380del | p.1461_1461del | 0.009236 | NM_004187 | frameshift deletion |
| P5 | relapse | KMT2C | c.2185A>G | p.N729D | 0.156 | NM_170606 | nonsynonymous SNV |
| P5 | relapse | KMT2C | c.2189C>A | p.S730Y | 0.0466 | NM_170606 | nonsynonymous SNV |
| P5 | relapse | KDM5C | c.4378_4380del | p.1461_1461del | 0.0122 | NM_004187 | frameshift deletion |
| P5 | relapse | RUNX1 | c.964_965del | p.S322fs | 0.4331 | NM_001754 | frameshift deletion |
| P6 | diagnosis | SF1 | c.1449_1451del | p.483_484del | 0.0081 | NM_201995 | frameshift deletion |
| P6 | diagnosis | KDM6B | c.3107C>T | p.P1036L | 0.0854 | NM_001080424 | nonsynonymous SNV |
| P6 | relapse | SF1 | c.1449_1451del | p.483_484del | 0.0195 | NM_201995 | frameshift deletion |
| P6 | relapse | BLM | c.1683_1685del | p.561_562del | 0.0152 | NM_000057 | frameshift deletion |
| P6 | relapse | ROBO3 | c.1745C>G | p.T582S | 0.0162 | NM_022370 | nonsynonymous SNV |
| P6 | relapse | SETD2 | c.1979A>G | p.N660S | 0.0397 | NM_014159 | nonsynonymous SNV |
| P6 | relapse | KDM6B | c.3107C>T | p.P1036L | 0.169 | NM_001080424 | nonsynonymous SNV |
| P6 | relapse | NT5C2 | c.712C>G | p.R238G | 0.1815 | NM_012229 | nonsynonymous SNV |
| P6 | relapse | TP53 | c.919+2_919+26del | c.919+2_919+26del | 0.2368 | NM_000546 | Splice_Site |
| P7 | diagnosis | NRAS | c.181C>A | p.Q61K | 0.0339 | NM_002524 | nonsynonymous SNV |
| P7 | diagnosis | PTPN11 | c.181G>A | p.D61N | 0.0519 | NM_002834 | nonsynonymous SNV |
| P7 | diagnosis | KRAS | c.35G>A | p.G12D | 0.0293 | NM_004985 | nonsynonymous SNV |
| P7 | diagnosis | KRAS | c.38G>A | p.G13D | 0.0095 | NM_004985 | nonsynonymous SNV |
| P7 | relapse | KRAS | c.38G>A | p.G13D | 0.2984 | NM_004985 | nonsynonymous SNV |
| P8 | diagnosis | KRAS | c.35G>C | p.G12A | 0.3417 | NM_004985 | nonsynonymous SNV |
| P8 | diagnosis | TRAF3 | c.598G>A | p.V200M | 0.3488 | NM_003300 | nonsynonymous SNV |
| P8 | relapse | KRAS | c.35G>C | p.G12A | 0.0821 | NM_004985 | nonsynonymous SNV |
| P8 | relapse | TRAF3 | c.598G>A | p.V200M | 0.1296 | NM_003300 | nonsynonymous SNV |
| P8 | relapse | KMT2D | c.6006_6051del | p.S2002fs | 0.0367 | NM_003482 | frameshift deletion |
| P8 | relapse | KMT2D | c.6053delT | p.I2018fs | 0.0477 | NM_003482 | frameshift deletion |
| P9 | diagnosis | KMT2C | c.2120T>C | p.I707T | 0.0107 | NM_170606 | nonsynonymous SNV |
| P9 | diagnosis | BCOR | c.2633T>C | p.V878A | 0.4852 | NM_001123383 | nonsynonymous SNV |
| P9 | diagnosis | TET2 | c.3730C>T | p.L1244F | 0.189 | NM_001127208 | nonsynonymous SNV |
| P9 | diagnosis | BCOR | c.G1645A | p.G549S | 0.5262 | NM_001123383 | nonsynonymous SNV |
| P9 | relapse | NT5C2 | c.1100G>A | p.R367Q | 0.0357 | NM_012229 | nonsynonymous SNV |
| P9 | relapse | PCLO | c.1456_1485del | p.486_495del | 0.0117 | NM_033026 | nonframeshift deletion |
| P9 | relapse | NR3C1 | c.1906C>G | p.L636V | 0.1523 | NM_000176 | nonsynonymous SNV |
| P9 | relapse | BCOR | c.2633T>C | p.V878A | 0.4397 | NM_001123383 | nonsynonymous SNV |
| P9 | relapse | NT5C2 | c.712C>G | p.R238G | 0.0363 | NM_012229 | nonsynonymous SNV |
| P9 | relapse | NT5C2 | c.713G>A | p.R238Q | 0.1696 | NM_012229 | nonsynonymous SNV |
| P9 | relapse | BCOR | c.G1645A | p.G549S | 0.5502 | NM_001123383 | nonsynonymous SNV |
| P10 | relapse | SRCAP | c.5607delC | p.G1869fs | 0.0141 | NM_006662 | frameshift deletion |
| P10 | relapse | IKZF1 | c.71_84del | p.P24fs | 0.0188 | NM_006060 | frameshift deletion |
| P10 | relapse | ABL1 | c.944C>T | p.T315I | 0.0353 | NM_005157 | nonsynonymous SNV |
| P11 | diagnosis | CDKN2A | c.157dupA | p.M53fs | 0.4466 | NM_000077 | frameshift insertion |
| P11 | diagnosis | SOX11 | c.673_675del | p.225_225del | 0.0845 | NM_003108 | frameshift deletion |
| P11 | diagnosis | EGR2 | c.925_927del | p.309_309del | 0.018 | NM_000399 | frameshift deletion |
| P11 | remission | SOX11 | c.673_675del | p.225_225del | 0.056 | NM_003108 | frameshift deletion |
| P11 | remission | EGR2 | c.925_927del | p.309_309del | 0.017 | NM_000399 | frameshift deletion |
| P11 | relapse | TRIM24 | c.130G>C | p.G44R | 0.4012 | NM_003852 | nonsynonymous SNV |
| P11 | relapse | PCLO | c.1508_1557del | p.Q503fs | 0.0101 | NM_033026 | nonframeshift deletion |
| P11 | relapse | CDKN2A | c.157dupA | p.M53fs | 0.4462 | NM_000077 | frameshift insertion |
| P11 | relapse | KMT2A | c.158C>T | p.A53V | 0.0948 | NM_001197104 | nonsynonymous SNV |
| P11 | relapse | NUMB | c.1889G>A | p.R630H | 0.0387 | NM_001005743 | nonsynonymous SNV |
| P11 | relapse | ETV6 | c.502_530del | p.N168fs | 0.3714 | NM_001987 | frameshift deletion |
| P11 | relapse | SOX11 | c.673_675del | p.225_225del | 0.0826 | NM_003108 | frameshift deletion |
| P11 | relapse | EGR2 | c.925_927del | p.309_309del | 0.0306 | NM_000399 | frameshift deletion |
| P12 | diagnosis | SOX11 | c.673_675del | p.225_225del | 0.0829 | NM_003108 | frameshift deletion |
| P12 | remission | SOX11 | c.673_675del | p.225_225del | 0.048 | NM_003108 | frameshift deletion |
| P12 | relapse | NT5C2 | c.1219G>C | p.D407H | 0.368 | NM_012229 | nonsynonymous SNV |
| P12 | relapse | SOX11 | c.673_675del | p.225_225del | 0.052 | NM_003108 | frameshift deletion |
| P13 | relapse | MACF1 | c.11330G>A | p.R3777H | 0.0106 | NM_012090 | nonsynonymous SNV |
| P13 | relapse | BCL11B | c.1388C>T | p.A463V | 0.0183 | NM_138576 | nonsynonymous SNV |
| P13 | relapse | TRIM24 | c.1405C>T | p.R469W | 0.0283 | NM_003852 | nonsynonymous SNV |
| P13 | relapse | NR3C1 | c.1430G>A | p.R477H | 0.0112 | NM_000176 | nonsynonymous SNV |
| P13 | relapse | STAG1 | c.1600C>T | p.R534C | 0.0154 | NM_005862 | nonsynonymous SNV |
| P13 | relapse | PDGFRA | c.1672C>T | p.R558C | 0.0333 | NM_006206 | nonsynonymous SNV |
| P13 | relapse | ROBO3 | c.1927G>A | p.V643I | 0.0134 | NM_022370 | nonsynonymous SNV |
| P13 | relapse | EZH2 | c.2036G>A | p.R679H | 0.0228 | NM_001203247 | nonsynonymous SNV |
| P13 | relapse | PDGFRB | c.2137G>A | p.A713T | 0.0117 | NM_002609 | nonsynonymous SNV |
| P13 | relapse | BCOR | c.221G>A | p.R74H | 0.0248 | NM_001123383 | nonsynonymous SNV |
| P13 | relapse | NF1 | c.2224G>A | p.A742T | 0.2356 | NM_000267 | nonsynonymous SNV |
| P13 | relapse | BCL11B | c.2593G>A | p.V865I | 0.0115 | NM_138576 | nonsynonymous SNV |
| P13 | relapse | NF1 | c.2999G>A | p.R1000H | 0.0452 | NM_000267 | nonsynonymous SNV |
| P13 | relapse | KMT2A | c.3248G>A | p.R1083Q | 0.013 | NM_001197104 | nonsynonymous SNV |
| P13 | relapse | TET2 | c.3602G>A | p.R1201H | 0.0113 | NM_001127208 | nonsynonymous SNV |
| P13 | relapse | ROBO3 | c.3887G>A | p.R1296Q | 0.0121 | NM_022370 | nonsynonymous SNV |
| P13 | relapse | CREBBP | c.4337G>A | p.R1446H | 0.0165 | NM_004380 | nonsynonymous SNV |
| P13 | relapse | TP53 | c.524G>A | p.R175H | 0.013 | NM_000546 | nonsynonymous SNV |
| P13 | relapse | CSMD1 | c.5381C>T | p.T1794M | 0.082 | NM_033225 | nonsynonymous SNV |
| P13 | relapse | KRAS | c.53C>T | p.A18V | 0.0505 | NM_004985 | nonsynonymous SNV |
| P13 | relapse | MACF1 | c.6317G>A | p.R2106H | 0.1034 | NM_012090 | nonsynonymous SNV |
| P13 | relapse | IL2RB | c.716A>G | p.D239G | 0.0995 | NM_001346223 | nonsynonymous SNV |
| P13 | relapse | MSH6 | c.718C>T | p.R240X | 0.797 | NM_000179 | stopgain |
| P13 | relapse | TP53 | c.743G>A | p.R248Q | 0.1265 | NM_000546 | nonsynonymous SNV |
| P13 | relapse | FAT1 | c.7960G>A | p.V2654I | 0.2461 | NM_005245 | nonsynonymous SNV |
| P13 | relapse | SRCAP | c.8210G>A | p.R2737Q | 0.0206 | NM_006662 | nonsynonymous SNV |
| P13 | relapse | ROBO1 | c.881G>A | p.R294Q | 0.1038 | NM_002941 | nonsynonymous SNV |
| P14 | relapse | NF1 | c.305T>G | p.M102R | 0.1009 | NM_000267 | nonsynonymous SNV |
| P14 | relapse | ABL1 | c.944C>T | p.T315I | 0.4134 | NM_005157 | nonsynonymous SNV |
| P15 | diagnosis | FAT1 | c.3770G>A | p.R1257Q | 0.5899 | NM_005245 | nonsynonymous SNV |
| P15 | diagnosis | KRAS | c.38G>A | p.G13D | 0.118 | NM_004985 | nonsynonymous SNV |
| P15 | relapse | JAK3 | c.2731C>T | p.R911C | 0.01 | NM_000215 | nonsynonymous SNV |
| P15 | relapse | FAT1 | c.3770G>A | p.R1257Q | 0.4998 | NM_005245 | nonsynonymous SNV |
| P16 | diagnosis | TP53 | c.743G>A | p.R248Q | 0.1977 | NM_000546 | nonsynonymous SNV |
| P16 | relapse | KMT2D | c.5867+2T>A | - | 0.0251 | NM_003482 | Splice_Site |
| P16 | relapse | TP53 | c.743G>A | p.R248Q | 0.2916 | NM_000546 | nonsynonymous SNV |
| P17 | diagnosis | FLT3 | c.1992G>C | p.M664I | 0.0119 | NM_004119 | nonsynonymous SNV |
| P17 | diagnosis | MSH6 | c.218A>C | p.N73T | 0.0154 | NM_000179 | nonframeshift deletion |
| P17 | diagnosis | PTPN11 | c.227A>G | p.E76G | 0.0301 | NM_002834 | nonsynonymous SNV |
| P17 | diagnosis | NRAS | c.436G>A | p.A146T | 0.0943 | NM_002524 | nonsynonymous SNV |
| P17 | diagnosis | FAT1 | c.7916C>G | p.S2639C | 0.0104 | NM_005245 | nonsynonymous SNV |
| P17 | relapse | NR3C1 | c.2231_2232insT | p.T744fs | 0.1839 | NM_000176 | frameshift insertion |
| P18 | diagnosis | PTPN11 | c.181G>C | p.D61H | 0.0294 | NM_002834 | nonsynonymous SNV |
| P18 | diagnosis | MAPK1 | c.20_22del | p.7_8del | 0.0915 | NM_002745 | nonframeshift deletion |
| P18 | diagnosis | PTPN11 | c.226G>A | p.E76K | 0.0366 | NM_002834 | nonsynonymous SNV |
| P18 | diagnosis | NR3C1 | c.283_284insG | p.T95fs | 0.0102 | NM_000176 | frameshift insertion |
| P18 | diagnosis | NSD2 | c.3295G>A | p.E1099K | 0.2201 | NM_133330 | nonsynonymous SNV |
| P18 | diagnosis | KRAS | c.437C>T | p.A146V | 0.2754 | NM_004985 | nonsynonymous SNV |
| P18 | relapse | PTPN11 | c.181G>C | p.D61H | 0.2158 | NM_002834 | nonsynonymous SNV |
| P18 | relapse | TP53 | c.845G>C | p.R282P | 0.4611 | NM_000546 | nonsynonymous SNV |
| P18 | relapse | RUNX1 | c.866G>C | p.G289A | 0.1486 | NM_001754 | nonsynonymous SNV |
| P19 | diagnosis | NRAS | c.183A>C | p.Q61H | 0.0124 | NM_002524 | nonsynonymous SNV |
| P19 | diagnosis | PTPN11 | c.227A>C | p.E76A | 0.0923 | NM_002834 | nonsynonymous SNV |
| P19 | diagnosis | TP53 | c.847C>A | p.R283S | 0.0983 | NM_000546 | nonsynonymous SNV |
| P19 | diagnosis | PAX5 | c.943T>G | p.Y315D | 0.0064 | NM_016734 | nonsynonymous SNV |
| P19 | remission | PAX5 | c.943T>G | p.Y315D | 0.0068 | NM_016734 | nonsynonymous SNV |
| P19 | relapse | CTNNB1 | c.1040dupT | p.L347fs | 0.1594 | NM_001904 | frameshift insertion |
| P19 | relapse | BCL11B | c.1168C>T | p.R390W | 0.1003 | NM_138576 | nonsynonymous SNV |
| P19 | relapse | BCL11B | c.1387G>A | p.A463T | 0.0085 | NM_138576 | nonsynonymous SNV |
| P19 | relapse | BCL11B | c.1697G>A | p.R566H | 0.101 | NM_138576 | nonsynonymous SNV |
| P19 | relapse | TNFRSF14 | c.185G>A | p.R62H | 0.0108 | NM_003820 | nonsynonymous SNV |
| P19 | relapse | PTPN11 | c.227A>C | p.E76A | 0.1761 | NM_002834 | nonsynonymous SNV |
| P19 | relapse | CUX1 | c.2281G>A | p.A761T | 0.0103 | NM_181552 | nonsynonymous SNV |
| P19 | relapse | MSH6 | c.2299A>G | p.T767A | 0.0084 | NM_000179 | nonsynonymous SNV |
| P19 | relapse | BCOR | c.3043T>C | p.Y1015H | 0.1918 | NM_001123383 | nonsynonymous SNV |
| P19 | relapse | NSD2 | c.3448A>T | p.T1150S | 0.0246 | NM_133330 | nonsynonymous SNV |
| P19 | relapse | ASXL1 | c.3818G>A | p.R1273H | 0.0167 | NM_015338 | nonsynonymous SNV |
| P19 | relapse | TET2 | c.4351C>T | p.R1451W | 0.0111 | NM_001127208 | nonsynonymous SNV |
| P19 | relapse | BCL11B | c.533C>T | p.P178L | 0.0121 | NM_138576 | nonsynonymous SNV |
| P19 | relapse | CREBBP | c.7297G>A | p.D2433N | 0.1208 | NM_004380 | nonsynonymous SNV |
| P19 | relapse | TP53 | c.847C>A | p.R283S | 0.1444 | NM_000546 | nonsynonymous SNV |
| P19 | relapse | PAX5 | c.943T>G | p.Y315D | 0.0265 | NM_016734 | nonsynonymous SNV |
| P20 | relapse | PRPS1 | c.216C>G | p.I72M | 0.0287 | NM_002764 | nonsynonymous SNV |
| P20 | relapse | NR3C1 | c.2247C>G | p.F749L | 0.031 | NM_000176 | nonsynonymous SNV |
| P20 | relapse | ABL1 | c.944C>T | p.T315I | 0.0548 | NM_005157 | nonsynonymous SNV |
| P21 | diagnosis | BLM | c.1683_1685del | p.561_562del | 0.0071 | NM_000057 | frameshift deletion |
| P21 | relapse | BCL6 | c.1191G>T | p.Q397H | 0.0346 | NM_001706 | nonsynonymous SNV |
| P21 | relapse | PCLO | c.14426A>G | p.D4809G | 0.0678 | NM_033026 | nonsynonymous SNV |
| P21 | relapse | PCLO | c.1508_1557del | p.Q503fs | 0.0104 | NM_033026 | nonframeshift deletion |
| P21 | relapse | TP53 | c.673-2A>G | c.673-2A>G | 0.1444 | NM_000546 | Splice_Site |
| P21 | relapse | BLM | c.1683_1685del | p.561_562del | 0.0174 | NM_000057 | frameshift deletion |
| P22 | remission | KDM5C | c.4378_4380del | p.1461_1461del | 0.00906 | NM_004187 | frameshift deletion |
| P22 | relapse | KMT2D | c.15339C>A | p.Y5113X | 0.0117 | NM_003482 | stopgain |
| P22 | relapse | KRAS | c.175G>A | p.A59T | 0.0113 | NM_004985 | nonsynonymous SNV |
| P22 | relapse | KRAS | c.179G>T | p.G60V | 0.0643 | NM_004985 | nonsynonymous SNV |
| P22 | relapse | KRAS | c.202A>T | p.R68W | 0.0193 | NM_004985 | nonsynonymous SNV |
| P22 | relapse | PTPN11 | c.205G>A | p.E69K | 0.0123 | NM_002834 | nonsynonymous SNV |
| P22 | relapse | PTPN11 | c.215C>T | p.A72V | 0.0104 | NM_002834 | nonsynonymous SNV |
| P22 | relapse | NRAS | c.35G>A | p.G12D | 0.0205 | NM_002524 | nonsynonymous SNV |
| P22 | relapse | KDM5C | c.4378_4380del | p.1461_1461del | 0.0131 | NM_004187 | frameshift deletion |
| P22 | relapse | USH2A | c.6014C>A | p.A2005D | 0.1084 | NM_206933 | nonsynonymous SNV |
| P22 | relapse | KRAS | c.68T>G | p.L23R | 0.0393 | NM_004985 | nonsynonymous SNV |
| P23 | diagnosis | SH2B3 | c.1192_1193insT | p.R398fs | 0.7311 | NM_005475 | frameshift insertion |
| P23 | diagnosis | EPOR | c.1278_1279insCCTGTTTTAC | p.T427fs | 0.0685 | NM_000121 | frameshift insertion |
| P23 | diagnosis | ANKRD26 | c.1736_1738del | p.579_580del | 0.011 | NM_001256053 | frameshift deletion |
| P23 | diagnosis | NF1 | c.2184_2185insCATACCT | p.V728fs | 0.0428 | NM_000267 | frameshift insertion |
| P23 | diagnosis | ARID1B | c.250_252del | p.84_84del | 0.021 | NM_017519 | frameshift deletion |
| P23 | diagnosis | KMT2A | c.52_54del | p.18_18del | 0.0176 | NM_001197104 | frameshift deletion |
| P23 | diagnosis | NF1 | c.5546+2T>C | - | 0.019 | NM_000267 | Splice_Site |
| P23 | remission | ANKRD26 | c.1736_1738del | p.579_580del | 0.0076 | NM_001256053 | frameshift deletion |
| P23 | remission | ARID1B | c.250_252del | p.84_84del | 0.019 | NM_017519 | frameshift deletion |
| P23 | remission | KMT2A | c.52_54del | p.18_18del | 0.00577 | NM_001197104 | frameshift deletion |
| P23 | relapse | SH2B3 | c.1192_1193insT | p.R398fs | 0.639 | NM_005475 | frameshift insertion |
| P23 | relapse | TP53 | c.140delC | p.P47fs | 0.0158 | NM_000546 | frameshift deletion |
| P23 | relapse | KMT2D | c.16520A>G | p.E5507G | 0.4111 | NM_003482 | nonsynonymous SNV |
| P23 | relapse | ANKRD26 | c.1736_1738del | p.579_580del | 0.0195 | NM_001256053 | frameshift deletion |
| P23 | relapse | ARID1B | c.250_252del | p.84_84del | 0.0325 | NM_017519 | frameshift deletion |
| P23 | relapse | NF1 | c.5546+2T>C | c.5546+2T>C | 0.4397 | NM_000267 | Splice_Site |
| P23 | relapse | TP53 | c.614A>G | p.Y205C | 0.1027 | NM_000546 | nonsynonymous SNV |
| P23 | relapse | TP53 | c.734G>A | p.G245D | 0.0404 | NM_000546 | nonsynonymous SNV |
| P24 | diagnosis | ABL1 | c.128G>T | p.S43I | 0.0312 | NM_005157 | nonsynonymous SNV |
| P24 | relapse | ABL1 | c.128G>T | p.S43I | 0.0136 | NM_005157 | nonsynonymous SNV |
